# Regulation of Glycerol-3-Phosphate Dehydrogenase by Hypoxia-Inducible Factor-1 in WSSV-Infected White Shrimp *Litopenaeus vannamei*

**DOI:** 10.3390/v18091020

**Published:** 2026-09-15

**Authors:** Jesús Alfredo Rosas-Rodríguez, Cesar Muñoz-Bacasehua, Guadalupe González-Ochoa, Enrico Alejandro Ruiz, Misael Daniel Mancilla-Morales, Luis Alberto Zamora-Alvarez, José Guadalupe Soñanez-Organis

**Affiliations:** 1 Departamento de Ciencias Químico-Biológicas y Agropecuarias, Facultad Interdisciplinaria de Ciencias Biológicas y de Salud, Universidad de Sonora, Navojoa 85880, Mexico; cesar.munoz@unison.mx (C.M.-B.); guadalupe.gonzalezochoa@unison.mx (G.G.-O.); luis.zamora@unison.mx (L.A.Z.-A.); 2 Departamento de Zoología, Escuela Nacional de Ciencias Biológicas, Instituto Politécnico Nacional, Colonia Santo Tomás, Ciudad de México 11340, Mexico; eruizc@ipn.mx; 3 Estación Científica La Malinche, Centro Tlaxcala de Biología de la Conducta, Universidad Autónoma de Tlaxcala, Tlaxcala 90000, Mexico; mdmancilla@uatx.mx

**Keywords:** white spot syndrome virus, gene expression, enzymatic activity, glycerol-3-phosphate dehydrogenase, gene silencing

## Abstract

Hypoxia-inducible factor-1 (HIF-1) induces the Warburg effect in the *Litopenaeus vannamei* shrimp infected with white spot syndrome virus (WSSV) to satisfy the energetic and biosynthetic demands required for viral replication. Glycerol-3-phosphate dehydrogenase (GPDH) is a key enzyme linking carbohydrate metabolism, lipid biosynthesis, and cellular redox balance; however, its role during WSSV infection remains poorly understood. In this study, the complete coding sequences of the cytoplasmic (LvGPDHc) and mitochondrial (LvGPDHm) GPDH isoforms were characterized from the hepatopancreas of *L. vannamei*, and their regulation by HIF-1α during WSSV infection was investigated. Sequence and phylogenetic analyses revealed that both proteins are highly conserved among crustaceans and contain the characteristic domains required for catalytic activity. WSSV infection decreases the mRNA expression of both GPDH isoforms and increases the cytoplasmic GPDH activity. In contrast, HIF-1α silencing increases the mRNA expression of both GPDH isoforms and increases the mitochondrial GPDH activity. The concentrations of glycerol-3-phosphate and citrate increase with WSSV infection and return to basal levels upon HIF-1α silencing. Results suggest that WSSV infection in white shrimp is associated with increased lipid biosynthesis via cytoplasmic GPDH, which contributes to viral replication, whereas mitochondrial GPDH may help maintain cellular redox status. Furthermore, these results demonstrate that the expression of GPDH isoforms is regulated by HIF-1, contributing to metabolic reprogramming in shrimp during WSSV infection.

## 1. Introduction

The glycerol-3-phosphate dehydrogenase (GPDH, E.C. 1.1.5.) plays an important role in lipid biosynthesis by converting dihydroxyacetone phosphate (DHAP) to glycerol-3-phosphate (G3P) and G3P back to DHAP [1]. GPDH is involved in maintaining the redox potential across the inner mitochondrial membrane [2] and provides a link between carbohydrate and lipid metabolism via the G3P shuttle [3]. There are 2 isoforms of GPDH, a cytoplasmic one (cGPDH EC 1.1.1.8), which is soluble and nicotinamide adenine dinucleotide (NAD)-dependent, and a mitochondrial one (mGPDH, EC 1.1.99.5), which is bound to the inner membrane and is flavin adenine dinucleotide (FAD)-dependent [4]. The cGPDH (E.C. 1.1.1.1.8) catalyzes the conversion of DHAP to G3P, and mGPDH (EC 1.1.5.3) catalyzes the conversion of G3P to DHAP [5]. The main role of these isoforms is the oxidation of cytosolic NADH produced by glycolysis and the reduction of FAD, a mechanism that takes place in the G3P shuttle.

The white spot syndrome virus (WSSV) induces the Warburg effect in the white shrimp *Litopenaeus vannamei*, an abnormal glycolytic response in which glucose consumption and lactate accumulation increase to meet the energy needs of the shrimp and the virus [6,7]. The Warburg effect increases glycolytic flux and the concentration of glycolytic intermediates such as glucose-6-phosphate (G6P) [8] and DHAP [9], which can be used to meet energy demand and provide essential raw materials for WSSV replication. In white shrimp, the WSSV-induced glycolysis is regulated by the hypoxia-inducible factor-1 (HIF-1) [7,8,10], a transcriptional factor formed by an oxygen-regulated α subunit (HIF-1α) and a constitutive β subunit (HIF-1β) [11]. However, it is currently unknown whether HIF-1 regulates the expression of the gene encoding GPDH isoforms during WWS infection.

In crustaceans, and even less so in white shrimp, there are few studies on the role of GPDH in the presence of pathogens. In the shrimp *Exopalaemon carinicauda*, WSSV decreases GPDH gene expression in the cephalothorax 12–27 h post-infection and increases the expression of dihydroxyacetone phosphate acyltransferase (DHAPAT), which catalyzes the synthesis of phospholipids from DHAP [12]. In white shrimp, only nucleotide sequences of GPDH isoforms are deposited in the GenBank database; however, their complementary DNA (cDNA) sequences have not been characterized. In this work, the complete coding cDNA sequences for GPDH isoforms were obtained from the hepatopancreas. Furthermore, we investigated the effects of silencing HIF-1α on the expression of both GPDH isoforms in WSSV-infected shrimp, as well as on cytoplasmic and mitochondrial GPDH activities and G3P and citrate concentrations, in order to better understand its role during WSSV infection.

## 2. Materials and Methods

### 2.1. Animal Handling, Silencing of HIF-1 and WSSV Infection

Healthy and specific-pathogen-free *L. vannamei* shrimp (15 ± 2 g) were acclimated for four weeks to laboratory conditions, kept in 200 L aquaria with controlled temperature (between 28 °C and 30 °C), constant aeration (6.02 mg/L), and salinity (35 ppt). To verify their specific-pathogen-free status, a representative sample of shrimp was screened for WSSV by polymerase chain reaction (PCR) according to the protocol described by Durand and Lightner (2002) [13]. Shrimp were fed 3% of their biomass with commercial feed pellets containing 35% protein (Purina). Water quality was maintained by constant recirculation through biological filters of 500 microns and UV radiation.

The experimental inoculum of WSSV and the double-stranded RNA (dsRNA) for HIF-1α were prepared as previously described [7,8,10]. Each experimental group was injected intramuscularly as follow: (1) saline solution (SS: TRIS-NaCl: 20 mM Tris; 400 mM NaCl; pH = 7.4) both as control (SS group, *n* = 10) and representing baseline levels, (2) 100 µL of WSSV inoculum (1.4 × 10^5^ copy number) (WSSV group, *n* = 30), and (3) 100 µL of WSSV inoculum (1.4 × 10^5^ copy number) and 15 µg of dsRNA HIF-1α (WSSV/dsRNA group, *n* = 30). Hepatopancreas was collected from five individual shrimp (*n* = 5) as follows: SS group, at 24 h after starting the experiment; WSSV group, at 24 and 48 h post-infection; and WSSV/dsRNA group, at 24 and 48 h post-treatment with HIF-1α dsRNA. Each hepatopancreas was processed separately and considered an independent biological replicate for subsequent analyses.

### 2.2. LvGPDHc and LvGPDHm Characterization

The 5′ and 3′ ends of each GPDH isoform were obtained from hepatopancreas using primers designed on their respective untranslated regions (UTRs) of each homolog nucleotide sequence of GPDH from white shrimp deposited in GenBank (Table 1). The following primers were used to obtain the 5′ and 3′ ends from: (1) GPDH cytoplasmic (LvGPDHc), LvGPDcF2 + LvGPDcR1 and LvGPDcF1 + LvGPDcR2, respectively; (2) GPDH mitochondrial (LvGPDHm), LvGPDmF2 + LvGPDmR1 and LvGPDmF1 + LvGPDmR2, respectively. The PCRs were performed using 25 μL of Platinum PCR SuperMix (Invitrogen, Carlsbad, CA, USA), 3 μL of cDNA and 1 μL (20 μM) of each primer were mixed and subjected to the following conditions: 75 °C for 5 min for one cycle; 94 °C for 3 min for one cycle; 40 cycles of 94 °C for 30 s, 58 °C for 40 s and 68 °C for 2 min; and an overextension step of 68 °C for 7 min. PCR products of 662 bp and 693 bp for LvGPDHc and 1019 bp and 1479 bp for LvGPDHm were obtained, cloned into the pGEM-T Easy Vector System (Promega, San Luis Obispo, CA, USA) and sequenced.

The nucleotide sequence of each LvGPDH isoform were analyzed as follows: the predicted amino acid sequence were obtained using a translation web site (https://web.expasy.org/translate/ (accessed on 1 August 2026)), aligned with other GPDH using Clustal Omega (https://www.ebi.ac.uk/jdispatcher/msa/clustalo (accessed on 1 August 2026)), compared with protein databases using BLAST (https://blast.ncbi.nlm.nih.gov/Blast.cgi; accessed on 1 August 2026), the identification of protein domains, conserved motives and catalytic residues was made with InterPro 86.0 (https://www.ebi.ac.uk/interpro/ (accessed on 1 August 2026)), and the signal peptides and protease recognition sites were analyzed with SignalP-5.0 (https://services.healthtech.dtu.dk/service.php?SignalP-5.0. (accessed on 1 August 2026)), and molecular weight and isoelectric point for the PK proteins were calculated with ExPASy (https://web.expasy.org/compute_pi/ (accessed on 1 August 2026)).

### 2.3. Phylogenetic Analysis and Homology Modeling

Deduced amino acid sequences of LvGPDH isoforms and other animals are available in GenBank-NCBI (Table 2), and they were analyzed in MEGA XI [14]. Amino acid sequence alignment was carried out with MUSCLE using UPGMA default parameters and allowing gaps. The best model of evolution was selected by maximum likelihood using a neighbor-joining tree. The phylogenetic reconstruction was performed by Maximum Likelihood (ML) using the amino acid LG [15] model with Gamma-distributed rates among sites, the Nearest-Neighbor-Interchange (NNI) heuristic method with an initial Bio NJ tree, and 1000 bootstrap pseudoreplicates. The outgroup in the cytoplasmic sequences was *Escherichia coli*, whereas in the mitochondrial sequences, it was *Deinococcus proteolyticus*.

### 2.4. Quantification of mRNA for GPDH Isoforms

Total RNA isolation from hepatopancreas, genomic DNA elimination, and cDNA synthesis were performed using TRIzol reagent (Invitrogen), DNase I digestion (Promega, Madison, WI, USA), and GoScript™ reverse transcriptase kit (Promega, Madison, WI, USA), respectively, following the manufacturer’s instructions. The mRNA for LvGPDHc and LvGPDHm was quantified by quantitative PCR and normalized to the expression of the ribosomal protein L8 using primer sequences shown in Table 1. Two PCRs were performed for each cDNA (2 data points per sample and 7 data points for each group) using a Step One Real-Time PCR System (Applied Biosystems, Foster City, CA, USA) in a final volume of 15 µL containing 7.5 µL of GoTaq qPCR Master Mix (Promega), 5 µL of Milli-Q water, 0.25 µL of each primer (20 µM) and 2 µL of cDNA (equivalent to 100 ng of total RNA). After a denaturation step at 95 °C for 10 min, amplification was performed for 40 cycles at 95 °C for 15 s and 63 °C for 1 min, with a single fluorescence measurement and a final melting curve program that increased by 0.3 °C every 20 s from 60 to 95 °C. Positive and negative controls were included for each gene. Standard curves were run for each gene and L8 ribosomal protein to determine amplification efficiency using dilutions of 5 × 10^−3^ to 5 × 10^−8^ ng μL^−1^ of PCR fragments.

### 2.5. Enzymatic Activity from GPDH Isoform

Proteins were prepared from 20 mg of hepatopancreas using the NE-PER^TM^ Nuclear and Cytoplasmic Extraction Reagents kit (Pierce, Rockford, IL, USA), and total protein concentration was measured using the Pierce™ BCA Protein Assay Kits (Pierce, Rockford, IL, USA). GPDH mitochondrial (EC 1.1.5.3) activity was determined using the methodology described by Pecinová et al. (2020) [16]. All reaction were measured in duplicate at a final volume of 200 µL in a 96-well plate, into which were placed 150 µL of the working solution (50 mM potassium chloride, 10 mM Tris-HCl, 1 mM EDTA, 1 mM KCN, and 1 mg/mL bovine serum albumin) at pH of 7.4, 15 µL of 2.6-dichlorophenolindophenol (DCPIP, 100 µM) and 20 µL of cytoplasmic protein extract diluted at 1:10 or control 15 µL of G3P (25 mM). The reaction was started by adding 15 µL of G3P (25 mM) and 15 µL of 2.6-dichlorophenolindophenol (DCPIP, 100 μM). Two reference controls were used: (1) the G3P control containing 150 µL of assay medium, 15 µL of G3P (25 mM), and 15 µL of DCPIP (100 µM), and (2) the sample control containing 150 µL of assay medium, 20 µL of a 1:10-diluted sample, and 15 µL of DCPIP (100 µM). Mitochondrial GPDH activity was determined spectrophotometrically as G3P: 2,6-dichlorophenolindophenol oxidoreductase (GP:DCPIP, ε610 = 20.1/mM/cm) and monitored at 610 nm for 10 min at 30 °C, monitoring the absorbance every 30 s using a Variouskan^TM^ LUX spectrophotometer (Thermo Fisher Scientific, Waltham, MA, USA). Enzymatic activity was expressed as Units/μg of protein^−1^. One mitochondrial GPDH unit is the amount of enzyme that oxidizes 1.0 μmol of DCPIP per minute at pH 7.4 and a temperature of 37 °C.

The GPDH cytoplasmic (EC 1.1.1.8) activity was measured using the Glycerol-3-Phosphate Dehydrogenase Activity Colorimetric Assay kit (SIGMA-ALDRICH), which detects the concentration of NADH based on absorbance at 450 nm, proportional to the enzymatic activity present. A standard curve was generated according to the manufacturer’s instructions, and enzyme activity was calculated from the rate of NADH production derived from the linear region of the NADH-versus-time plots. All measurements were performed in duplicate, measuring the absorbance of each sample at 450 nm in a Variouskan^TM^ LUX spectrophotometer (Thermo Fisher Scientific, Waltham, MA, USA). Enzymatic activity was expressed as Units/μg of protein^−1^. One cytoplasmic GPDH unit is the amount of enzyme that generates 1.0 μmol of NADH per minute at pH 8 and a temperature of 37 °C.

### 2.6. Citrate and G3P

Citrate and G3P concentrations were measured using the Citrate Assay Kit (SIGMA-ALDRICH, Burlington, MA, USA) and Glycerol 3-Phosphate Colorimetric Assay Kit (SIGMA-ALDRICH, Burlington, MA, USA), respectively, following the manufacturer’s instructions. All measurements were performed in triplicate, measuring the absorbance of each sample at 570 nm and 450 nm for citrate and G3P, respectively, in a Varioskan LUX multimode microplate reader (Thermo Fisher Scientific, Waltham, MA, USA). Results are expressed as nmol μL^−1^ of citrate or G3P μg protein^−1^.

### 2.7. Statistics

Data were tested for normality and homogeneity of variance, and differences between groups were detected by one-way ANOVA with a Bonferroni post hoc test, using the saline solution-injected shrimps as control. Means (± s.e.m.) were considered statistically different at *p* < 0.05 (STATISTICAL 8 software, StatSoft Inc., Tulsa, OK, USA).

## 3. Results

### 3.1. cDNA Characterization of LvGPDH Isoforms

The full-length LvGPDHc (GenBank SUB14694088) and LvGPDHm (GenBank SUB14694087) cDNA sequences for *L. vannamei* were obtained from shrimp hepatopancreas. The LvBADHc sequence is 1152 bp long with a 1095 bp open reading frame (ORF) and encodes a predicted protein of 365 amino acid residues and a calculated molecular weight of 40 kDa. The translated LvGPDHc exhibited high identity to GPDH cytoplasmic from the Chinese white shrimp *P. chinensis* (97%), the Japanese shrimp *P. japonicus* (96%), the gazami crab *P. trituberculatus* (85%), and the green mud crab *S. paramamosain* (84%). The LvGPDHm sequence is 2282 bp long with a 2241 bp ORF and encodes a predicted protein of 747 amino acid residues and a calculated molecular weight of 83 kDa. The predicted protein LvGPDHm has identity to GPDH mitochondrial from the Indian prawn *Penaeus indicus*, the giant tiger prawn *P. monodon*, and the Chinese white shrimp *P. chinensis* with 99%, the lobster *H. americanus* (89%), and the gazami crab *P. trituberculatus* (86%).

**Figure 1:**
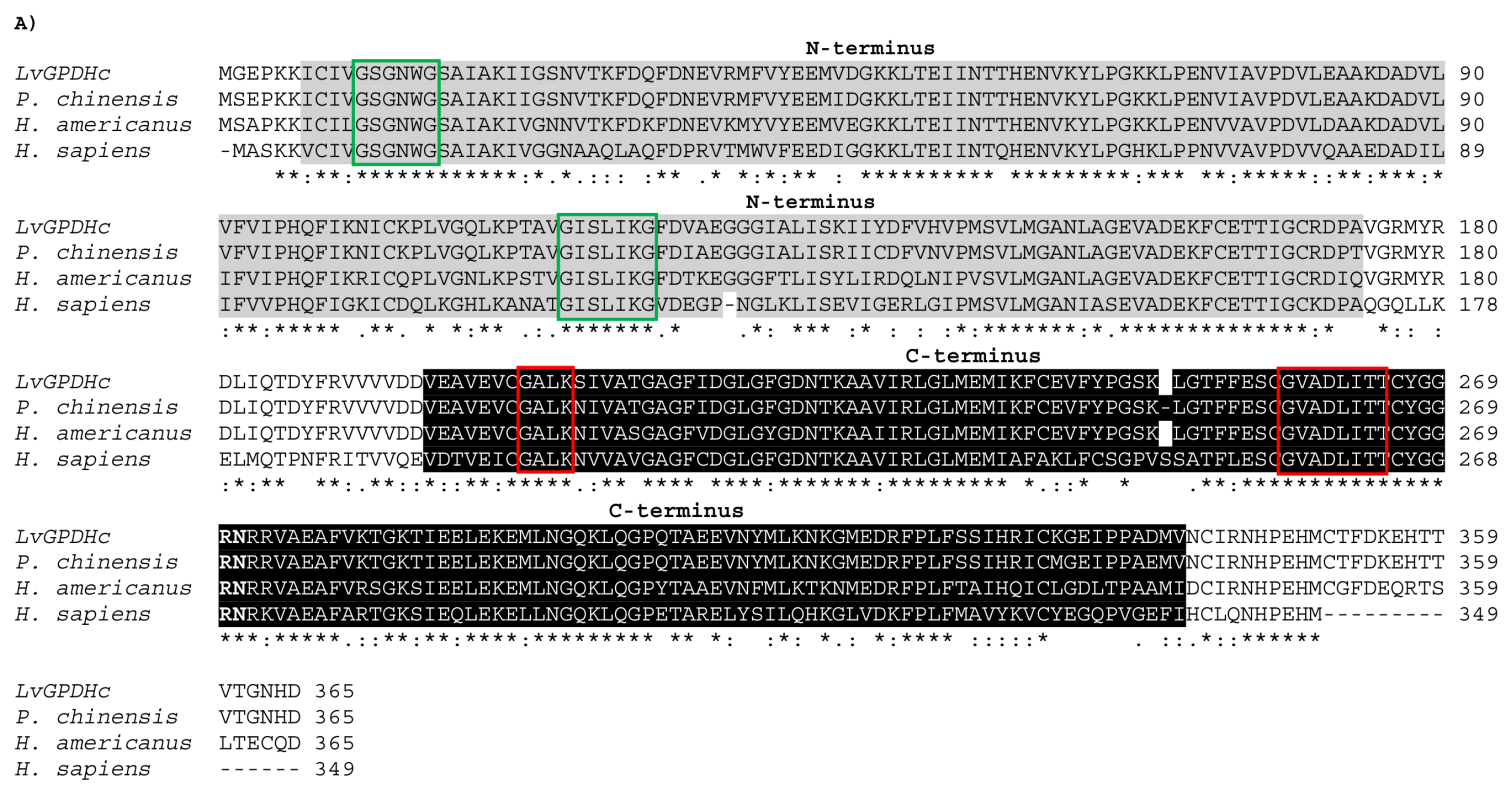

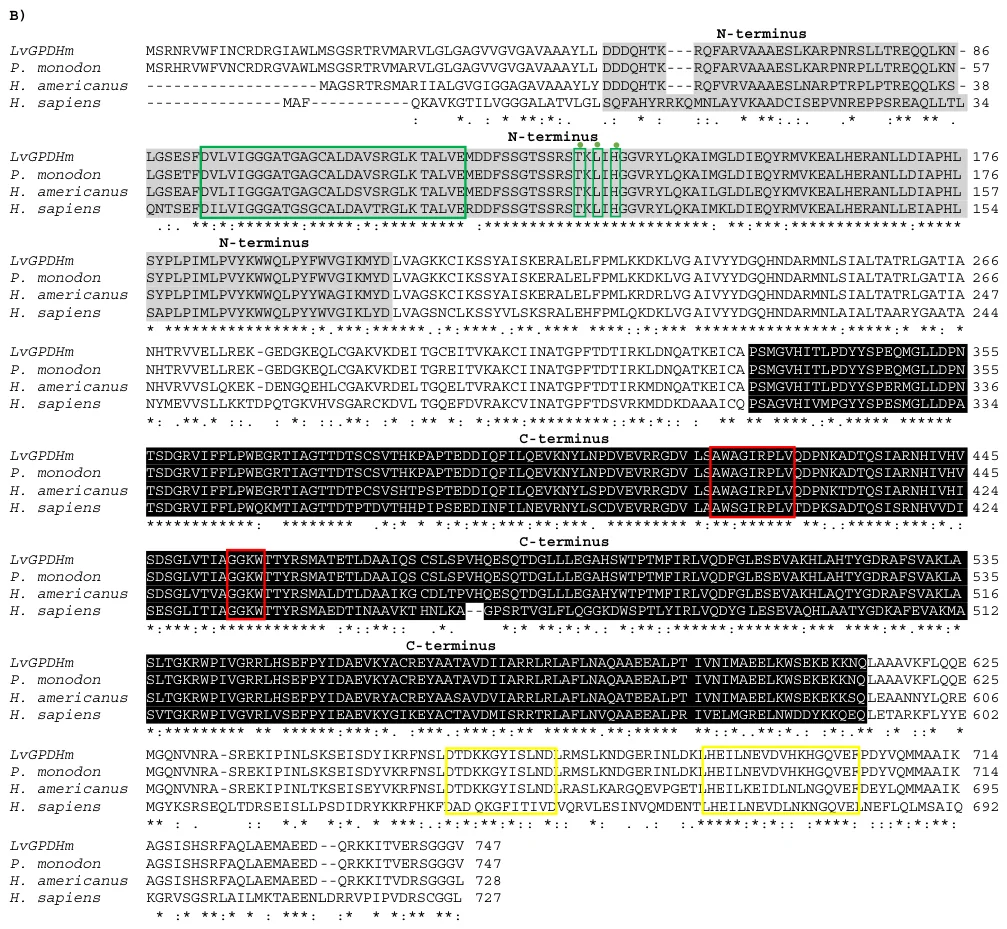
Multiple sequence alignment of LvGPDHc (**A**) and LvGPDHm (**B**) sequences. The predicted amino acid sequences of GPDH from *L. vannamei*, *P. monodon*, *H. americanus*, and *H. sapiens* are included in the analysis. (*) identical residues; (:) conservative substitutions; and (.) semi-conservative substitutions. The region and residues involved in the binding site for substrate, NAD or FAD, and Ca (^2+^)-binding are shown in red, green, and yellow solid boxes, respectively.

Both amino acid sequences of LvGPDH isoforms present residues highly conserved for their coenzyme (NAD or FAD) and substrate binding (Figure 1). The following binding sites were identified for LvGPDHc: (1) substrate includes the residues ^203^GALK^206^ and ^258^GVADLITT^265^; and (2) NAD, ^11^GSGNWG^16^ and ^116^GISLIKG^122^ (Figure 1A); for LvGPDHm: (1) substrate includes the residues ^418^AWAGIRPLV^426^ and ^455^GGKW^458^; FAD, ^93^DVLVIGGGATGAGCALDAVSRGLKTALVE^121^ and ^134^TKLIH^138^; and Ca-(^2+^)-binding ^658^DTDKKGYISLND^669^ and ^686^LHEILNEVDVHKHGQ^702^ (Figure 1B).

### 3.2. Phylogenetic Analysis

**Figure 2:**
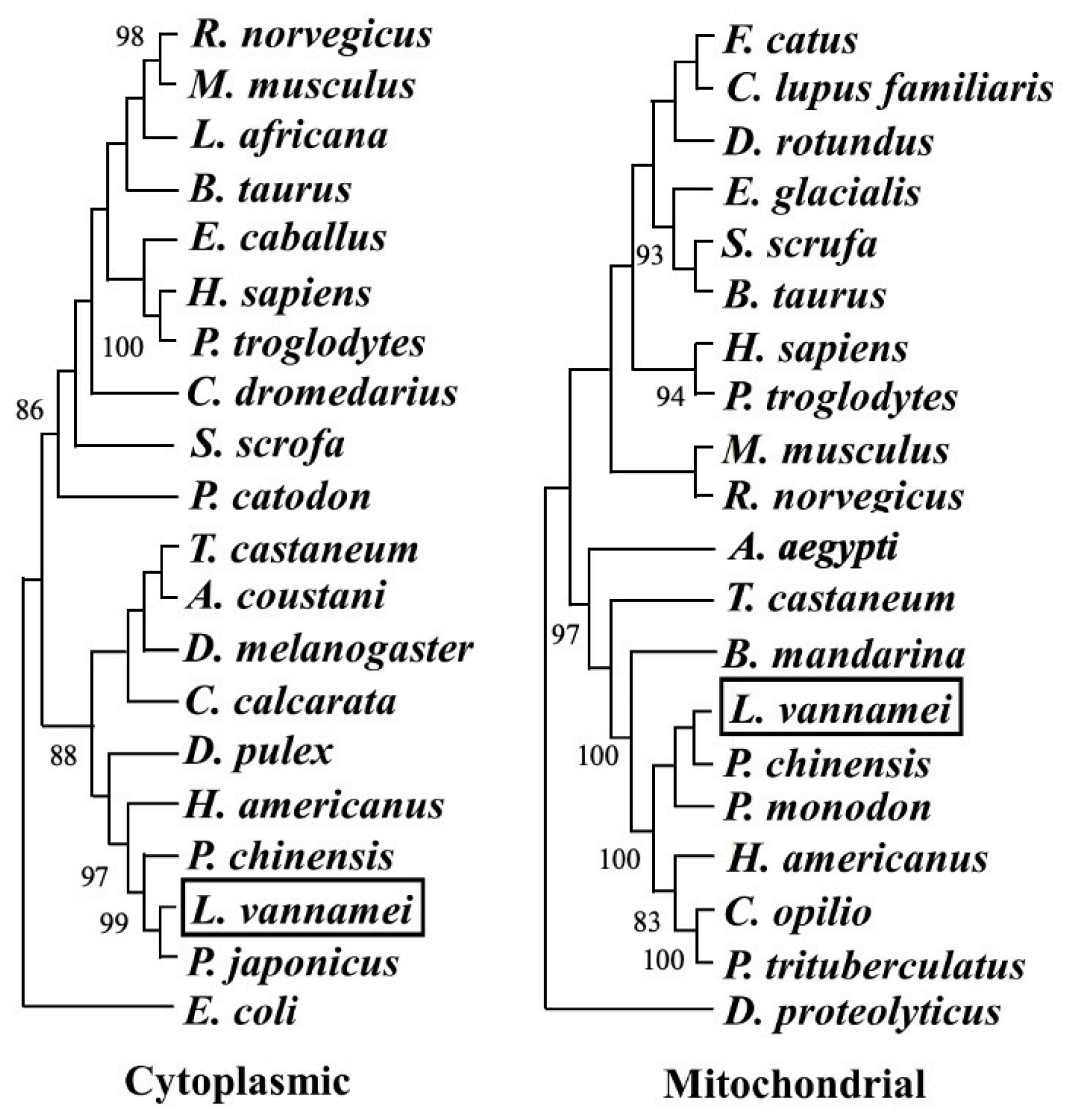
Consensus tree resulting from phylogenetic analysis of GPDH deduced amino acid sequences. The tree was obtained using the neighbor-joining method. Numbers at the base of each node indicate the percentages of bootstrap support based on 1000 bootstrap pseudoreplicates. The GenBank accession numbers for each GPDH sequence are included in Table 2.

Phylogenetic trees were constructed to analyze evolutionary relationships among several species, including LvGPDHc and LvGPDHm deduced amino acid sequences, using two types of genetic markers: cytoplasmic and mitochondrial (Figure 2). The phylogenetic tree based on cytoplasmic sequences (Figure 2, left) revealed the clustering of LvGPDHc with *P. japonicus*, showing a close evolutionary relationship within a clade that includes another decapod (*H. americanus* and *P. chinensis*), with a bootstrap support of 97%. Other well-supported clades include mammals such as *R. norvegicus*, *M. musculus*, and *L. africana*, with a bootstrap support of 98%.

Phylogenetic analysis with mitochondrial markers (Figure 2, right) placed LvGPDHm in a clade with *P. chinensis* and *P. monodon*, with a strong bootstrap support of 100%. This clade is closely related to other crustaceans such as *H. americanus* and *C. opilio.* In addition, a well-defined clade grouping mammals, including *F. catus* and *C. lupus familiaris*, was observed with a bootstrap support of 93%.

### 3.3. LvGPDHc and LvGPDHm Are Regulated Differentially by WSSV Infection

**Figure 3:**
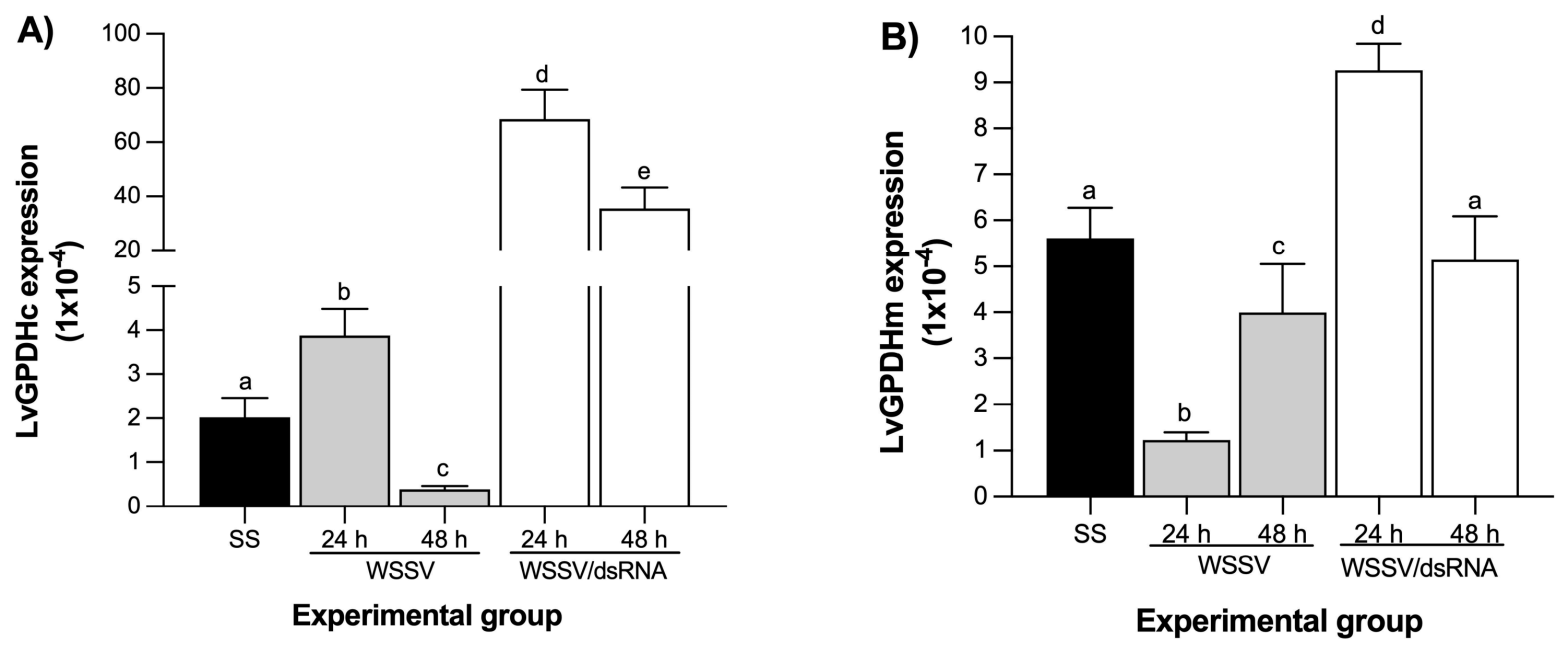
LvGPDHc (**A**) and LvGPDHm (**B**) expression levels in the hepatopancreas of shrimp infected with WSSV and silenced HIF-1α. The letters represent significant differences between the experimental groups (*p* > 0.05). SS: injected with saline solution; WSSV: infected group; WSSV/dsRNA: infected group with silenced HIF-1α.

The mRNA levels for LvGPDHc increased 1.9-fold in the hepatopancreas of the WSSV group at 24 h post-infection (hpi) compared to the control, while at 48 hpi they decreased 5.2-fold. In the WSSV/dsRNA group, LvGPDHc mRNA levels increased 33.8- and 17-fold at 24 and 48 h post-treatment (hpt), compared to the control. On the other hand, mRNA levels for LvGPDHc increased 17.6- and 175.5-fold in the WSSV/dsRNA group at 24 hpt compared to 24 and 48 hpi with WSSV, respectively, while in the WSSV/dsRNA group at 48 hpt increased 8.9- and 88.6-fold compared to 24 and 48 hpi with the WSSV, respectively (Figure 3A).

The mRNA levels for LvGPDHm decreased 4.6- and 1.4-fold in the hepatopancreas of the WSSV group at 24 and 48 hpi, respectively, compared to the control. In the WSSV/dsRNA group, LvGPDHm levels increased 1.7-fold at 24 hpt compared to the control, while at 48 hpt they returned to the basal level. The mRNA levels for LvGPDHm increased 7.5- and 2.3-fold in the WSSV/dsRNA group at 24 hpt compared to 24 and 48 hpi with WSSV, respectively (Figure 3B).

### 3.4. Cytoplasmic and Mitochondrial GPDH Activities

**Figure 4:**
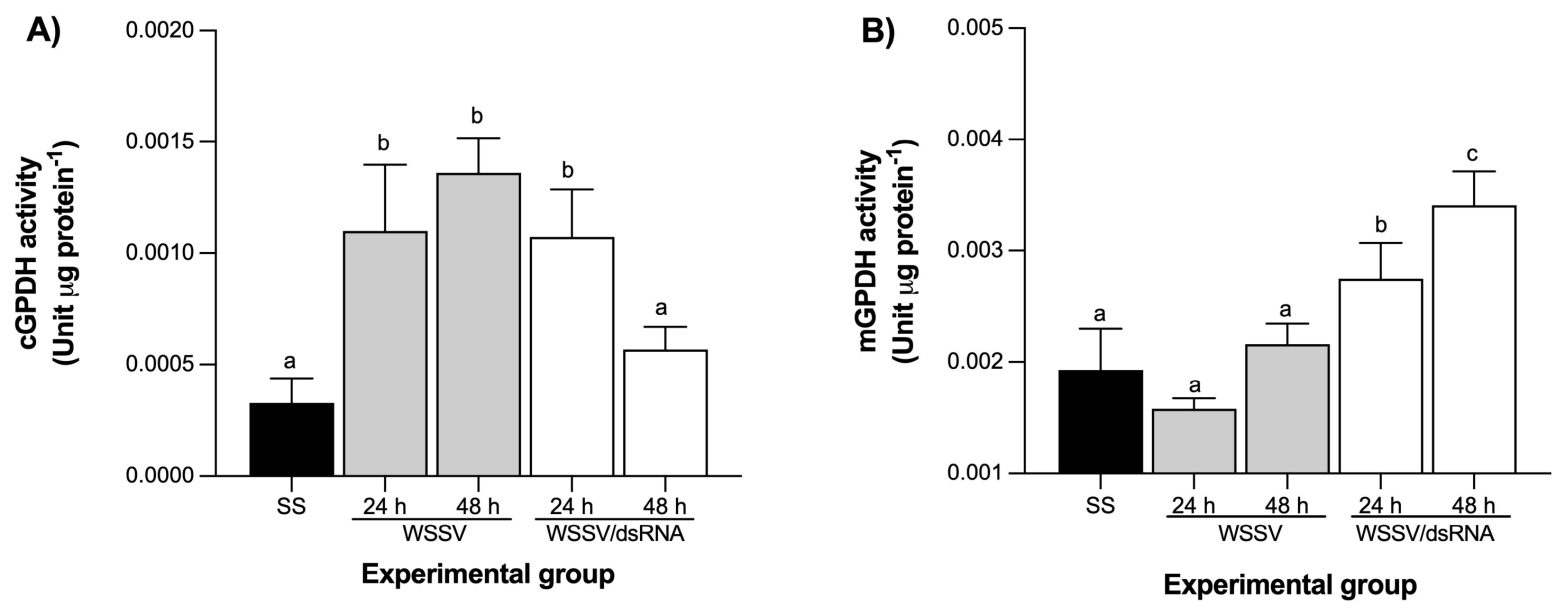
Cytoplasmic (**A**) and mitochondrial (**B**) GPDH activities in the hepatopancreas of white shrimp infected with WSSV and HIF-1α silenced. The letters represent significant differences between the experimental groups (*p* > 0.05). SS: injected with saline solution; WSSV: infected group; WSSV/dsRNA: infected group with silenced HIF-1α.

The cytoplasmic GPDH activity increased 3.4- and 5-fold in the hepatopancreas of the WSSV group at 24 and 48 hpi, respectively, with respect to the control. In the WSSV/dsRNA group, cGPDH activity increased 3.3-fold at 24 hpt relative to the control, while at 48 h it returned to basal levels. On the other hand, there was no change in cytoplasmic GPDH activity between the WSSV group at 24 and 48 hpi and the WSSV/dsRNA group at 24 hpt. In contrast, the cytoplasmic GPDH activity in the WSSV/dsRNA group at 48 hpt decreased 1.95- and 2.9-fold with respect to the WSSV group at 24 and 48 hpi, respectively, and 1.9-fold with respect to the WSSV/dsRNA group at 48 hpt (Figure 4A).

Mitochondrial GPDH activity did not show significant changes between the WSSV group at 24 and 48 hpi compared to the control, while in the WSSV/dsRNA group, it increased 1.4- and 1.8-fold at 24 and 48 hpt, respectively. On the other hand, mitochondrial GPDH activity increased 1.7- and 1.6-fold in the WSSV/dsRNA group at 24 and 48 hpt compared to the WSSV group at 24 and 48 hpi, respectively. Additionally, mitochondrial GPDH activity in the WSSV/dsRNA group at 48 hpt increased 1.3-fold compared to 24 hpt (Figure 4B).

### 3.5. G3P and Citrate Levels

**Figure 5:**
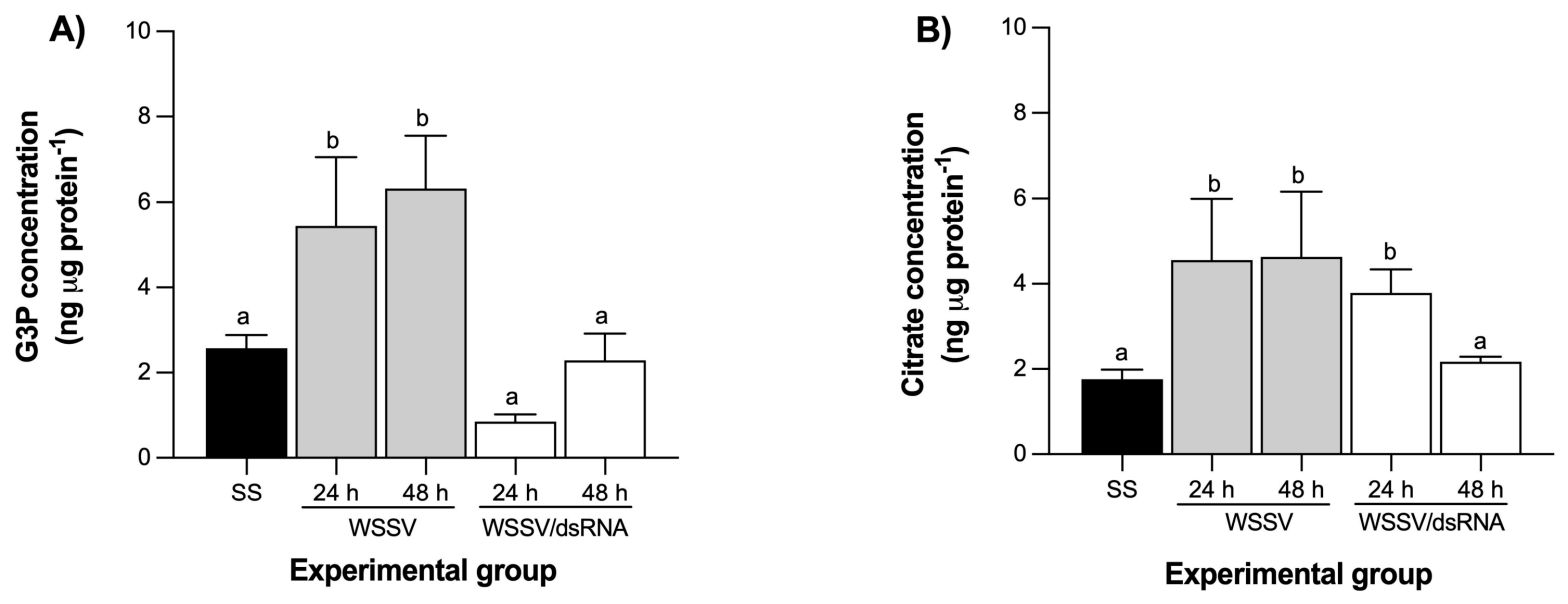
G3P (**A**) and Citrate (**B**) concentrations in the hepatopancreas of white shrimp infected with WSSV and HIF-1α silenced. The letters represent significant differences between each of the experimental groups (*p* > 0.05). SS: injected with saline solution; WSSV: infected group; WSSV/dsRNA: infected group with silenced HIF-1α.

The G3P levels increased 2.1- and 2.5-fold in the hepatopancreas of the WSSV group at 24 and 48 hpi, respectively, with respect to the control, while in the WSSV/dsRNA group, it returned to basal levels (Figure 5A). The citrate level increased 2.6-fold in the hepatopancreas of the WSSV group at 24 and 48 hpi, with respect to the control. In contrast, citrate level in the WSSV/dsRNA group increased 2.1-fold compared to the control, while at 48 h it returned to basal levels (Figure 5B).

## 4. Discussion

We have identified and characterized the coding region of the GPDH isoforms -LvGPDHc and LvGPDHm- of the hepatopancreas from white shrimp and demonstrated that both are differentially regulated during WSSV infection by HIF-1. Both shrimp GPDH amino acid sequences are highly similar to those in crustaceans, including conserved amino acid residues responsible for substrate and cofactor binding, indicating that these enzymes likely perform functions similar to those in other organisms [17]. Phylogenetic analyses demonstrated that both LvGPDHc and GPDHm are closely related to other GPDH crustaceans, including shrimp, highlighting their fundamental role in cellular metabolism. Previous studies have demonstrated that WSSV infection induces the Warburg effect in white shrimp, which is characterized by high glucose consumption, increased glycolytic flux, and the accumulation of glycolytic intermediates necessary for WSSV replication [6,7,8]. Furthermore, Chen et al. [6] showed that these metabolic changes are highly dynamic during infection, with distinct metabolic phases occurring between the early and late stages of WSSV replication. Consistent with this temporal metabolic reprogramming, LvGPDHc expression in the present study increased at 24 hpi but decreased at 48 hpi, suggesting that the regulation of this enzyme changes as infection progresses. All these metabolic changes are regulated by HIF-1, which acts as a central transcriptional regulator of energy metabolism in WSSV-infected white shrimp [7,8]. Because the G3P shuttle is an alternative pathway linking glucose metabolism to lipid biosynthesis and redox homeostasis, the regulation of LvGPDH isoforms represents a key mechanism through which WSSV redirects host resources toward viral production.

Mitochondrial GPDH oxidizes G3P to DHP and transfers reducing equivalents to the respiratory chain through the G3P shuttle [1,4]. The reduction in LvGPDHm transcript and mitochondrial GPDH activity, along with G3P accumulation during WSSV infection, suggests that suppression of this pathway may favor triacylglycerol and phospholipid synthesis. These are processes that viruses frequently exploit to generate membranes required for replication and virion assembly [18,19,20]. Therefore, a decrease in mitochondrial GPDH activity could help redirect carbon flux toward anabolic pathways that promote WSSV replication. In contrast, WSSV infection increased cytoplasmic GPDH activity despite a decrease in LvGPDHc transcript levels during infection. Similar differences between transcript abundance and enzyme activity have been reported in several host–pathogen interactions. They may result from post-transcriptional regulation, protein stabilization, substrate availability, or post-translational modifications [21,22]. Although the use of purified mitochondrial and cytoplasmic fractions would allow a more precise assessment of compartment-specific GPDH activities, the enzyme assays employed in this study were designed to evaluate functional changes associated with WSSV infection. The lack of a direct correlation between transcript abundance and enzyme activity may reflect post-transcriptional and post-translational regulatory mechanisms, as well as the distinct metabolic roles of cytoplasmatic and mitochondrial GPDH during the host response to viral infection. The increase in cytoplasmic GPDH activity and G3P concentration implies increased conversion of DHAP to G3P. During the Warburg effect induced by WSSV, DHAP is accumulated [9]; therefore, it could be utilized in lipid synthesis, thereby providing the structural components necessary for viral replication.

HIF-1α silencing increased the expression of both LvGPDH isoforms but increased the mitochondrial GPDH activity. Previous studies demonstrated that HIF-1 activation is required for the glycolytic reprogramming induced by WSSV, whereas HIF-1α silencing reduces glycolytic gene expression, glucose consumption, and viral replication [7,8,10]. Consequently, changes in LvGPDH expression, GPDH activity, G3P concentrations, and citrate accumulation are likely secondary consequences of diminished glycolytic carbon flow rather than primary targets of HIF-1 regulation. Because G3P production depends directly on glycolytic intermediates, inhibition of HIF-1 reduces the metabolic input required to sustain lipogenesis and other anabolic pathways associated with viral replication. The increase in LvGPDHm expression after HIF-1α silencing suggests that HIF-1 negatively regulates components of the G3P shuttle during infection. Such regulation may help maintain elevated levels of G3P available for anabolic processes during viral proliferation. Interestingly, HIF-1α silencing also reversed the accumulation of both G3P and citrate observed in infected shrimp. Citrate is a major source of cytosolic acetyl-CoA for fatty acid synthesis and has been identified as a key metabolite supporting membrane biogenesis in rapidly proliferating cells and virus-infected tissues [23,24,25,26]. The accumulation of G3P and citrate during WSSV infection suggests the activation of lipogenic pathways, while their reduction by HIF-1α silencing demonstrates that HIF-1-regulated glycolysis decreased in both flux and rate. Together with the normalization of G3P concentrations, these findings indicate that inhibition of HIF-1 disrupts the metabolic environment required for efficient viral replication.

The coordinated behavior of the two GPDH isoforms provides new insight into their physiological functions in infected shrimp. Cytoplasmic GPDH appears to primarily support G3P production for lipid biosynthesis, whereas mitochondrial GPDH may contribute to cellular redox balance through the G3P shuttle. During WSSV infection, activation of the cytoplasmic branch combined with repression of the mitochondrial branch would maximize the availability of G3P for phospholipid production. Conversely, restoration of mitochondrial GPDH activity after HIF-1α silencing may increase G3P oxidation and reduce substrate availability for lipid biosynthesis. This mechanism may contribute to the lower viral loads previously reported in shrimp with suppressed HIF-1 [10].

Our results are consistent with other host–pathogen systems in which GPDH metabolism contributes to microbial virulence and pathogen replication. GPDH is involved in glycerol metabolism, redox regulation, and pathogenicity in fungi and bacteria [27,28,29,30,31,32]. The deletion of GPDH affects the growth of the bacterium *Pseudomonas aeruginosa* due to a limitation in glycerol content [27,28]. In the fungus *Pyricularia oryzae*, cGPDH is involved in nitric oxide production, conidiation, and the utilization of various carbon sources such as pyruvate. On the other hand, mitochondrial GPDH is essential for glycerol utilization and fungal development, demonstrating that both isoforms are essential for virulence [32]. Studies in macrophages from mice infected with bacterial lipopolysaccharides show increased gene expression and enzymatic activity of mitochondrial GPDH [29]. Therefore, this study demonstrates that WSSV induces transcriptional and enzymatic components of the G3P shuttle through a HIF-1-dependent mechanism. Collectively, these results show that GPDH isoforms play a key role in linking glycolysis, lipid metabolism, and redox regulation during viral infection in white shrimp. Although our results strongly support the involvement of GPDH isoforms in WSSV-induced metabolic reprogramming, direct functional evidence linking these enzymes to viral replication remains to be established. Future studies using isoform-specific gene silencing or overexpression approaches will help clarify the precise contribution of each GPDH isoform during WSSV infection.

## 5. Conclusions

The full-length coding sequences of the LvGPDHc and LvGPDHm isoforms contain the domains necessary for their function and regulation and are differentially expressed during WSSV infection. WSSV infection increases cytoplasmic GPDH activity, decreases mitochondrial GPDH activity, and results in the accumulation of G3P and citrate. These changes, combined with the role of the hepatopancreas as an energy reservoir and in lipid biosynthesis, suggest that both GPDH isoforms function in a coordinated manner to restore the redox state and energy availability disrupted by WSSV. Reversal of these effects following HIF-1α silencing indicates that HIF-1 is a central regulator of G3P metabolism during WSSV infection. Therefore, enzymes associated with this pathway represent potential targets for strategies aimed at limiting WSSV propagation in shrimp aquaculture. Therefore, although the results support the involvement of GPDH in the metabolic reprogramming induced by WSSV, a causal role in viral replication remains to be demonstrated. Future studies using isoform-specific gene silencing or overexpression approaches will help clarify the precise contribution of each GPDH isoform during WSSV infection.

## Figures and Tables

**Table 1:** Primers used for the cDNA characterization and qPCR.

Primer Name	Nucleotide Sequences (5’-3’)	GenBank Accession Number
*LvGPDHc*		XM_027372168.1 XM_027366150.1
*LvGPDcF1*	CCAATGTCAGTCCTTATGGG	
*LvGPDcR1*	CCATCAATAAATCCAGCACC	
*LvGPDcF2*	CTAGCCAACATGGGTGAACC	
*LvGPDcR2*	CTTCATCAGTCGTGGTTGCC	
*LvGPDHm*		
*LvGPDmF1*	GCTTGGTGCTACTATTGCC	
*LvGPDmR1*	GGACACCCATACTTGGTGC	
*LvGPDmF2*	GCGAATCTTCGTTAGGAAAG	
*LvGPDmR2*	GGCTCAGGTCTACTGCTAG	
*L8*		DQ316258.1
*L8F*	GCTGAGGGCATGTACTCTGG	
*L8R*	CCTGAAGGGAGCTTTACACG	

**Table 2:** Amino acid sequences of cytoplasmic and mitochondrial GPDH were included in the phylogenetic analyses.

Organism	GenBank Accession Number	Organism	GenBank Accession Number
*Cytoplasmic GPDH*		*Mitochondrial GPDH*	
*Anopheles coustani*	XP_058123928.1	*Aedes aegypti*	XP_001664284.1
*Bos taurus*	NP_001030431.1	*Bombyx mandarina*	XP_028034134.1
*Camelus dromedarius*	KAB1270654.1	*B. taurus*	NP_001093766.1
*Ceratina calcarata*	XP_017889258.1	*Canis lupus familiaris*	XP_038302700.1
*Crassostrea angulate*	XP_052710948.1	*Chionoecetes opilio*	KAG0711285.1
*Daphnia pulex*	XP_046453440.1	*Desmodus rotundus*	XP_053774659.1
*Drosophila melanogaster*	NP_476567.1	*D. melanogaster*	AAG22263.1
*Equus caballus*	XP_001504291.1	*Eubalaena glacialis*	XP_061056334.1
*Homo sapiens*	NP_005267.2	*Felis catus*	XP_003990833.2
*Homarus americanus*	XP_042234676.1	*H. americanus*	XP_042236186.1
*Loxodonta africana*	XP_064140317.1	*H. sapiens*	P43304.3
*Mus musculus*	NP_034401.1	*M. musculus*	NP_001139292.1
*Pan troglodytes*	XP_001154939.1	*Pan troglodytes*	XP_063647286.1
*Penaeus chinensis*	XP_047471896.1	*P. chinensis*	XP_047469902.1
*Penaeus japonicus*	XP_042879330.1	*Penaeus monodon*	XP_037780674.1
*Physeter catodon*	XP_007107529.2	*Portunus trituberculatus*	XP_045135587.1
*Rattus norvegicus*	NP_071551.2	*R. norvegicus*	XP_063139207.1
*Sus scrofa*	NP_001177169.1	*Sus scrofa*	XP_020931436.1
*Tribolium castaneum*	XP_975007.2	*T. castaneum*	XP_008194241.1
*Escherichia coli*	KYL38306.1	*Deinococcus proteolyticus*	ADY26258.1

## Data Availability

The original contributions presented in this study are included in the article. Further inquiries can be directed to the corresponding authors.
